# GEISIR: gadolinium exposure induced systemic inflammatory response in dialysis patients

**DOI:** 10.1186/1532-429X-11-S1-P144

**Published:** 2009-01-28

**Authors:** Henning Steen, Constanze Merten, Dirk Lossnitzer, Stephanie Lehrke, Vedat Schwenger, Hugo A Katus, Evangelos Giannitsis

**Affiliations:** University of Heidelberg, Heidelberg, Germany

**Keywords:** Lymphopenia, Nephrogenic Systemic Fibrosis, Anuria, Late Sequela, Observational Series

## Introduction and purpose

Several late sequelae of the administration of gadolinium (Gd)-containing MRI contrast agents have been described in patients with advanced renal failure. In an observational series we found a remarkable frequency of peracute reactions after administration of Gd-DTPA used for cardiovascular evaluation before renal transplantation.

## Methods

In a 26 months observational period 13 of 136 hemodialysed or CAPD patients exhibited onset of fever, chills and nausea within hours after administration of Gd-DTPA peracute. A minority showed persistent cessation of residual diuresis. We performed blood cultures in most patients and evaluated white blood cell counts (WBC), eosinophils, CRP, heart rate and blood pressure.

## Results

Within an average of 12 h (range 12–36 h) after Gd-administration, the 13 patients (9 male, 4 female; median age 61 years, range 47 – 79) developed consistent symptomatology with fever (median 39.0°C, range 37.5 – 39.5), chills, malaise, hypotension, vomiting, dyspnea – initially raising suspicion of septicaemia. Subsequent blood cultures on bacterial contamination of the injected product remained negative throughout; bacterial or endotoxin contamination of the reagent was excluded. Steroids were tried in the first two patients without noticeable effect. In all subsequent patients symptoms were attenuated during the first 5 h dialysis (F60HPS with 280 ml/min blood flow) and disappeared within 72 h. CRP levels remained markedly elevated up to 14 days. Lymphopenia was seen in all patients and polymorphic neutrophils (PMN) remained normal. Two polyuric patients developed persistent anuria. After a median of 16 months none of these patients developed nephrogenic systemic fibrosis.

## Conclusion

This series with unusually severe acute phase reactions was caused by one specific preparation. Such peracute reactions may be relevant for the so far largely unresolved pathogenesis of the skin reaction to some Gd products in end-stage renal disease (ESRD) patients. It remains unresolved, whether the reaction observed with Gd-DTPA do in principle also occur with other Gd reagents.

